# Immunological Pattern in IgA Nephropathy

**DOI:** 10.3390/ijms21041389

**Published:** 2020-02-18

**Authors:** Clara Esteve Cols, Freddzia-Amanda Graterol Torres, Bibiana Quirant Sánchez, Helena Marco Rusiñol, Maruja Isabel Navarro Díaz, Jordi Ara del Rey, Eva Mª Martínez Cáceres

**Affiliations:** 1 Immunology Division, LCMN, Germans Trias i Pujol University Hospital and Research Institute, FOCIS Center of Excellence—UAB-Barcelona, 08916 Badalona, Spain; cesteve@cli.cat (C.E.C.); bquirant.germanstrias@gencat.cat (B.Q.S.); 2 Department of Cell Biology, Physiology and Immunology, Universitat Autònoma de Barcelona, 08193 Bellaterra, Spain; 3 Nephrology Division, Germans Trias i Pujol University Hospital, 08916 Badalona, Spainhmarco.germanstrias@gencat.cat (H.M.R.); minavarro.germanstrias@gencat.cat (M.I.N.D.)

**Keywords:** IgA Nephropathy, monocytes, CD89, biomarkers

## Abstract

The current gold-standard diagnostic technique for IgA nephropathy (IgAN), the leading form of primary glomerulonephritis, is renal biopsy. CD89 (the main IgA receptor) is expressed on the surface of monocytes and plays a role in disease pathogenesis. Immunocomplexes formed by sCD89 (soluble form) and Gd-IgA1 are related to disease prognosis. We hypothesize that reduced CD89 surface expression on monocytes may be a marker of disease severity. We aimed to analyze leukocyte subpopulations in peripheral blood and CD89 surface expression on monocytes in a prospective study of 22 patients and 12 healthy subjects (HS). Leukocyte subpopulations and CD89 expression were analyzed by flow cytometry. IgAN patients had a higher percentage of activated and effector memory CD4^+^ and CD8^+^ T lymphocytes, a lower percentage of transitional B lymphocytes and plasmablasts, and a higher percentage of CD56^dim^CD16^+^ NK cells and myeloid dendritic cells compared with HS. Correlations between reduced CD89 expression levels on nonclassical monocytes, histological findings of a poor prognosis on renal biopsy and baseline renal function were observed. IgAN patients show a characteristic immunological pattern in peripheral blood. A reduced expression level of CD89 on nonclassical monocytes identifies patients with a worse renal prognosis.

## 1. Introduction

IgA nephropathy (IgAN), the leading primary glomerulonephritis worldwide, is a significant cause of renal disease, leading to end-stage renal disease (ESRD) in up to 40% of patients about 30–40 years after diagnosis [1,2,3,4]. Clinical variability determines different disease courses and treatment remains a challenge. Many baseline prognostic factors have been described— glomerulosclerosis and tubulointerstitial fibrosis, lower glomerular filtration rate (GFR), nephrotic proteinuria and systolic blood pressure [5], while some serum biomarkers are known to be disease predictors.

The diagnosis of IgAN remains biopsy-proven, based on pathological criteria, including mesangial IgA deposits identified by direct immunofluorescence. However, in the last decade, many techniques have become available to help establish diagnostic suspicion and define the diagnosis in biopsies reported as nonspecific or where immunofluorescence cannot be performed.

The central finding of the physiopathogenic process in patients with IgAN is the presence of degalactosylated IgA1 (Gd-IgA1) [6]. This abnormal molecule (Gd-IgA1) may induce the generation of autoantibodies, inducing immune complexes formation. These complexes (composed of Gd-IgA1 and IgG linked the o-glycan hinge region of Gd-IgA1 [6,7]), are deposited in the glomerular mesangium, causing complement activation and renal damage. However, circulating immune complexes of Gd-IgA1 and soluble CD89 (sCD89) have also been found [7,8]. CD89 is the main IgA receptor involved in its functions and is expressed on myeloid cells, mainly monocytes. Gd-IgA1 molecules tend to aggregate, forming polymeric molecules [9]. These circulating polymeric Gd-IgA1 molecules induce the shedding of the extracellular domain of CD89, and Gd-IgA1-CD89 immune complexes. 

Although high levels of Gd-IgA1 may suggest the disease, they do not explain the appearance of nephropathy [10,11]. In fact, Gd-IgA1 levels in relatives of IgAN patients and in healthy subjects without renal disease have also been found. For this reason, new research focuses on the search of biomarkers to help to understand the physiopathogenic process.

Gd-IgA1 receptors in blood and renal tissue have been postulated as potential markers. Mesangial CD71 (transferrin receptor) is overexpressed in patients with IgA nephropathy, colocalizing with Gd-IgA1 deposits. Several studies have identified CD71 as a key receptor for binding polymeric Gd-IgA1- and Gd-IgA1-containing immune complexes [12,13], which cause mesangial cell activation and the release of inflammatory cytokines [4,8]. IL-1, TGF-β and other cytokines are leading factors in the physiopathogenic process of IgAN and are related to the severity of renal involvement. Several other molecules have been implicated in immune complex binding by mesangial cells (cytokines, such as IL-6 and TGF-β) [11]. Until now, no analysis of the characteristics of the different leucocyte subpopulations in peripheral blood and expression of CD89 on monocyte surfaces has been carried out in IgA nephropathy patients.

The aim of this study was to characterize the leukocyte subpopulation profile in the peripheral blood in IgAN patients, focusing on CD89 expression on monocytes.

## 2. Results

### 2.1. Immunophenotype of Leukocyte Subpopulations

IgAN patients had a higher absolute number of lymphocytes compared with healthy subjects (HS) (Patients: 2258 ± 253.7 lymphocytes/µL; HS: 1773 ± 80.71 lymphocyte/µL; p = 0.0209).

#### 2.1.1. T lymphocyte Subpopulations

The percentage of T lymphocytes (CD3^+^) was similar between IgAN patients and HS (Patients: 76.71 ± 1.428% CD3^+^; HS: 76.77 ± 0.9037% CD3^+^; p = 0.9685). Percentage of CD8^+^ and CD4^+^ T lymphocytes were lower in IgAN patients (23.37 ± 1.754% CD8^+^; 47.94 ± 2.027% CD4^+^) compared with HS (31.33 ± 1.126% CD8^+^; 62.28 ± 1.368% CD4^+^; p = 0.0003 and p < 0.0001, respectively). No significant differences were observed in the percentage of double-positive and double negative T lymphocytes between IgAN patients and HS (Figure S1a,b).

##### CD8^+^ T Lymphocytes

**Figure 1:**
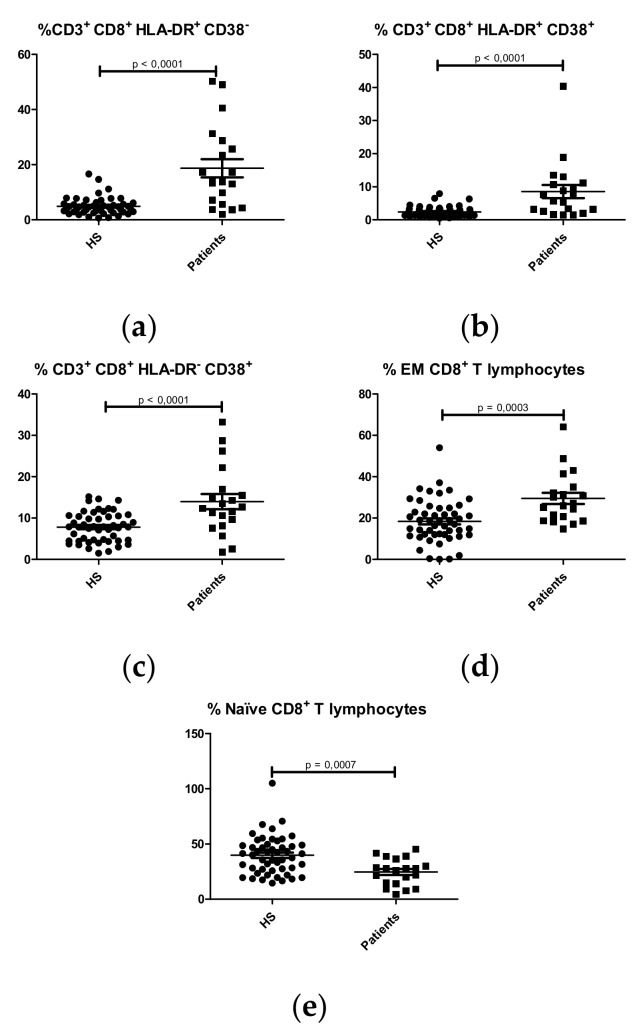
Comparison of percentages of CD8^+^ T lymphocyte subpopulations between HS (N = 50) and IgA nephropathy patients (N = 22). (**a**) Activated CD8^+^ T lymphocytes (CD3^+^CD8^+^HLA-DR^+^CD38^-^); (**b**) Activated CD8^+^ T lymphocytes (CD3^+^CD8^+^HLA-DR^+^CD38^+^); (**c**) Activated CD8^+^ T lymphocytes (CD3^+^CD8^+^HLA-DR^-^CD38^+^); (**d**) Effector Memory (EM) CD8^+^ T lymphocytes (CD3^+^CD8^+^CCR7^-^CD45RA^-^); (**e**) Naïve CD8^+^ T lymphocytes (CD3^+^CD8^+^CCR7^+^CD45RA^+^).

Higher percentages of activated (HLA-DR^+^CD38^-^, HLA-DR^+^CD38^+^, HLA-DR^-^CD38^+^) (Figure 1a–c) and effector memory (CCR7^-^CD45RA^-^) CD8^+^ T lymphocytes (Figure 1d) were observed in IgAN patients compared with HS. IgAN patients had lower percentages of naïve (CCR7^+^CD45RA^+^) T CD8^+^ lymphocytes than HS (Figure 1e). No significant differences were observed in central memory CD8^+^ T lymphocytes between patients and HS (Figure S1c).

##### CD4^+^ T lymphocytes

**Figure 2:**
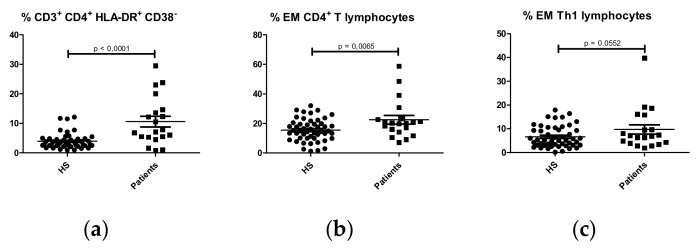
Comparison of percentages of CD4^+^ T lymphocyte subpopulations in HS (N = 50) and IgA nephropathy patients (N = 22). (**a**) Activated CD4^+^ T lymphocytes (CD3^+^CD4^+^HLA-DR^+^CD38^-^); (**b**) Effector Memory (EM) CD4^+^ T lymphocytes (CD3^+^CD4^+^CCR7^-^CD45RA^-^); (**c**) Effector Memory (EM) Th1 lymphocytes (CD3^+^CD4^+^CXCR3^+^CCR6^-^CCR7^-^CD45RA).

Higher percentages of activated (HLA-DR^+^CD38^-^) (Figure 2a) and effector memory (CCR7^-^CD45RA^-^) CD4^+^ T lymphocytes (Figure 2b) were found in IgAN patients compared with HS. Effector memory CD4^+^ T lymphocytes were classified as Th1 (CXCR3^+^CCR6^-^), Th2 (CXCR3^-^CCR6^-^) and Th17 (CXCR3^-^CCR6^+^). Higher percentages of effector memory Th1 cells were observed in IgAN patients compared with HS (Figure 2c). No significant differences were observed in effector memory Th2 and Th17 (Figure S1d,e) or naïve (Figure S1f) or central memory CD4^+^ T lymphocytes (Figure S1g–i).

No significant differences were observed in regulatory T lymphocytes between IgAN patients and HS. (Figure S1j).

#### 2.1.2. B lymphocyte Subpopulations

**Figure 3:**
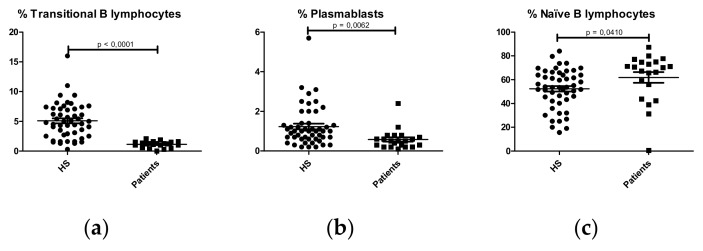
Comparison of percentages of B lymphocyte subpopulations in HC (N = 30) and IgA nephropathy patients (N = 22). (**a**) Transitional B lymphocytes (CD19^+^CD24^high^CD38^high^); (**b**) Plasmablasts (CD19^+^CD20^-^CD38^high^); (**c**) Naïve B lymphocytes (CD19^+^CD27^-^IgD^+^IgM^+^).

The percentage of B lymphocytes (CD19^+^) was similar between IgAN patients and HS (Patients: 10.86 ± 1.633% CD19+; HS: 10.80 ± 0.5160; p = 0.9657). However, analysis of B lymphocyte subpopulations showed that IgAN patients had lower percentages of transitional B lymphocytes (CD19^+^CD27^-^CD24^high^CD38^high^) (Figure 3a) and plasmablasts (CD19^+^CD27^+^CD20^-^CD38^high^) (Figure 3b), with higher percentages of naïve B lymphocytes (CD19^+^CD27^-^IgD^+^IgM^+^) (Figure 3c) compared with HS. No significant differences were observed in unswitched (CD19^+^CD27^+^IgD^+^IgM^+^; CD19^+^CD27^+^IgD^-^IgM^+^) (Figure S1k,l) and switched (CD19^+^CD27^+^IgD^-^IgM^-^) memory B lymphocytes (Figure S1m).

#### 2.1.3. Dendritic Cell Subpopulations

**Figure 4:**
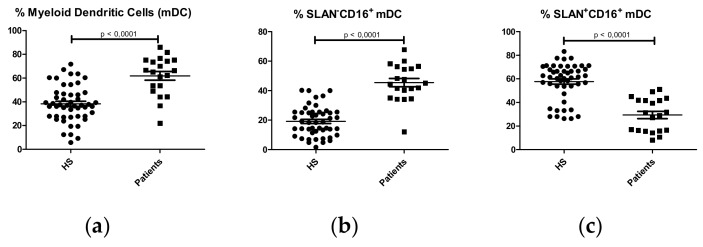
Percentages of dendritic cell subpopulations in HS (N = 50) and IgA nephropathy patients (N = 22). (**a**) Myeloid dendritic cells (mDC); (**b**) SLAN^-^CD16^+^ mDC; (**c**) SLAN^-^CD16^+^ mDC.

Negative selection of lymphocyte and monocyte surface markers (lineage^-^) and HLA-DR surface expression were used to characterize dendritic cells (DCs) (CD3^-^CD19^-^CD56^-^CD14^-^HLA-DR^+^). The percentage of DCs was higher in IgAN patients compared with HS (Patients: 9.755 ± 0.8972 %; HS:4.713 ± 0.5279 %; p < 0.0001). Deeper analysis of DC subsets showed a higher percentage of myeloid dendritic cells (HLA-DR^+^CD11c^+^CD123^-^) (Figure 4a), predominantly SLAN^-^CD16^+^ (Figure 4b,c) in IgAN patients. No significant differences were observed in plasmacytoid dendritic cells (HLADR^+^CD11c^-^CD123^+^) between IgAN patients and HS. (Figure S1n).

#### 2.1.4. NK Cell Subpopulations

**Figure 5:**
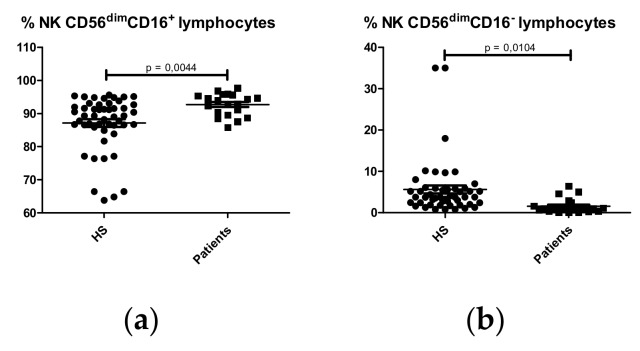
Percentages of natural killer (NK) lymphocyte subpopulations in HS (N = 50) and IgA nephropathy patients (N = 22). (**a**) NK CD56^dim^CD16^+^ lymphocytes; (**b**) NK CD56^dim^CD16^-^ lymphocytes.

Analysis of NK subpopulations showed a higher percentage of CD56^dim^CD16^+^ NK cells (Figure 5a) and a lower percentage of CD56^dim^CD16^-^ NK cells (Figure 5b) in IgAN patients compared with HS. However, no differences were observed in the percentage of CD56^bright^CD16^-^ NK cells (Figure S1o) or in total NK (CD56^dim^CD16^+^) cells between IgAN patients and HS (Patients: 8.610 ± 0.8671% NK lymphocytes; HS: 8.103 ± 0.4413% NK lymphocytes. p = 0.5691).

### 2.2. Characterization of Monocyte Subpopulations

IgAN patients had lower percentages of classical monocytes (CD14^+^CD16^-^) compared with HS (Patients 74.93 ± 1.542%; HS: 82.02 ± 1.711%; p = 0.0063) (Figure S2a). No significant differences were observed in intermediate (CD14^+^CD16^+^) (Patients: 13.62 ± 1.727%; HS: 11.50 ± 1.656%; p = 0.4223) or nonclassical monocyte (CD14^low^CD16^++^) percentages (Patients: 3.129 ± 0.346%; HS: 2.783 ± 0.489%; p = 0.5618) (Figure S2b,c). No differences in the absolute number of monocytes were observed compared with HS (Patients: 574 ± 51.47 monocytes/µL; HS: 472.3 ± 44.96 monocytes/µL; p = 0.1584)

**Figure 6:**
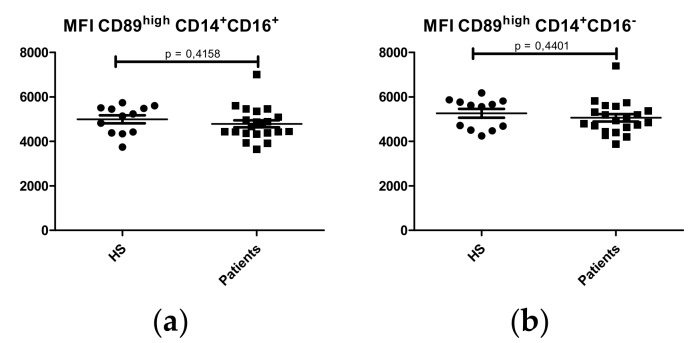
CD89^high^ Mean Fluorescence Intensity (MFI) analysis on monocytes subsets between IgAN patients (N = 22) and HS (N = 12). (**a**) Classical (CD14^+^CD16^-^) monocytes; (**b**) Intermediate (CD14^+^CD16^+^) monocytes.

To evaluate the relationship between CD89 expression levels and its release in a soluble form according to the physiopathology of IgAN, we analyzed CD89 MFI (Mean Fluorescence Intensity) on the surface of the three monocyte subpopulations. No significant differences in CD89 MFI on classical (CD14^+^CD16^-^) monocytes (Figure 6a) (Patients: 5061 ± 164.2 MFI; HS: 5260 ± 193.9 MFI; p = 0.4401) and intermediate (CD14^+^CD16^+^) monocytes (Figure 6b) (Patients: 4789 ± 160.4 MFI; HS: 4990 ± 183.3 MFI; p= 0.4158) were observed.

**Figure 7:**
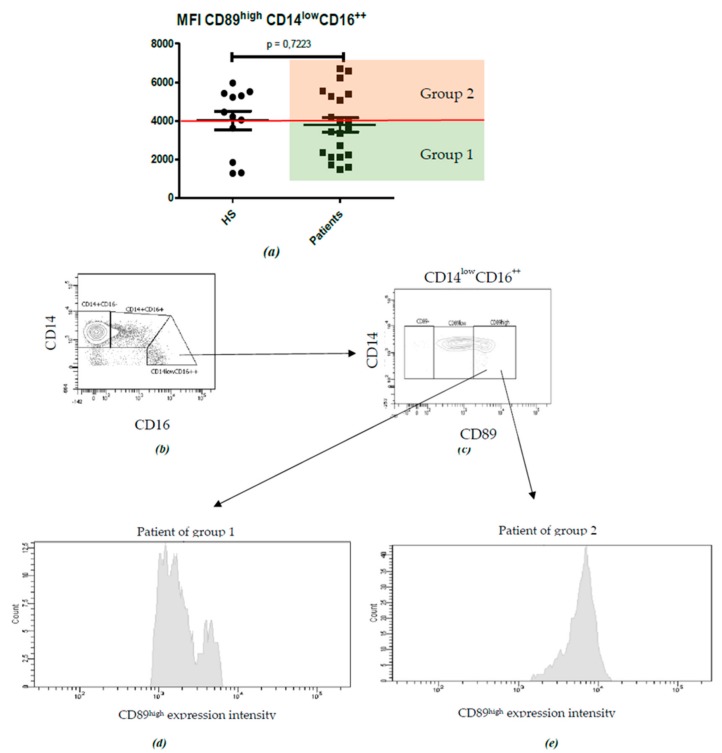
CD89^high^ MFI on nonclassical monocytes (CD14^low^CD16^++^) allows for distinguishing of two groups of patients. (**a**) Statistical analysis of CD89^high^ MFI on nonclassical monocytes (CD14^low^CD16^++^) between IgAN patients (N = 22) and HS (N = 12); (**b**) Monocytes were subdivided depending on CD14 vs. CD16 surface expression in classical monocytes (CD14^+^CD16^-^), intermediate monocytes (CD14^+^CD16^+^) and nonclassical monocytes (CD14^low^CD16^++^). (**c**) From the nonclassical monocytes (CD14^low^CD16^++^), three subpopulations were subdivided depending on the MFI of CD89: CD89-, CD89low and CD89 high. CD89^high^ nonclassical monocytes allowed for the differentiation of two groups of patients: (**d**) Group 1 (CD89^high^ MFI < 4000) and (**e**) Group 2 (CD89^high^ MFI > 4000).

When analyzing the nonclassical (CD14^-^CD16^++^) monocytes, although no significant differences were found (Patients: 3794 ± 372.3 MFI; HS: 4014 ± 485.1 MFI; p = 0.7223) (Figure 7a), we observed that patients’ distributions according to CD89 expression levels were not as homogeneous as it was on classical and intermediate monocytes. Further analysis (see Figure 7b–e) allowed us to distinguish two groups of patients according to CD89 MFI, with an area under the curve (AUC) of 0.5476 (95% CI 0.3313-0.7639) and a cut-off of 4000 (sensitivity 57.14%, specificity 66.67%) (Figure 7 and Figure S3), which allowed us to divide IgAN patients into two groups—group 1 patients with CD89 MFI < 4000, and group 2 patients with CD89 MFI > 4000.

**Figure 8:**
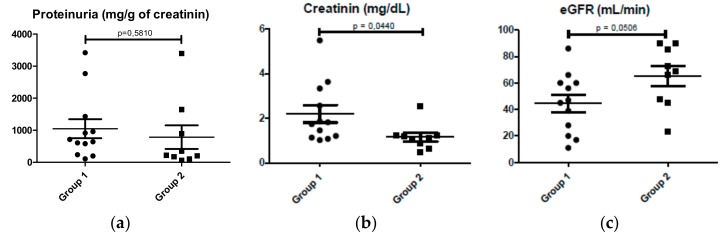
Comparison of renal function analysis between group 1 (N = 13) and group 2 (N = 9) of patients. (**a**) Grade of proteinuria; (**b**) serum creatinine levels; (**c**) renal glomerular filtrate.

Patients of group 1 had poor renal function with higher serum creatinine levels (1.157 ± 0.193 mg/dL in group 2 patients; 2.198 ± 0.389 mg/dL in group 1 patients; p = 0.0440), lower eGFR (65.44 ± 7.621 mL/min of group 2 patients; 44.58 ± 6.505 mL/min of group 1 patients; p = 0.0506) (Figure 8b–d) and a more severe renal biopsy, with more segmental glomerulosclerosis and interstitial fibrosis/tubular atrophy (Table 1). No significant differences in proteinuria were observed (785.4 ± 368.6 mg/g of creatinine in group 2 patients; 1049 ± 297.7 mg/L in group 1 patients; p = 0.5810) (Figure 8b).

**Figure 9:**
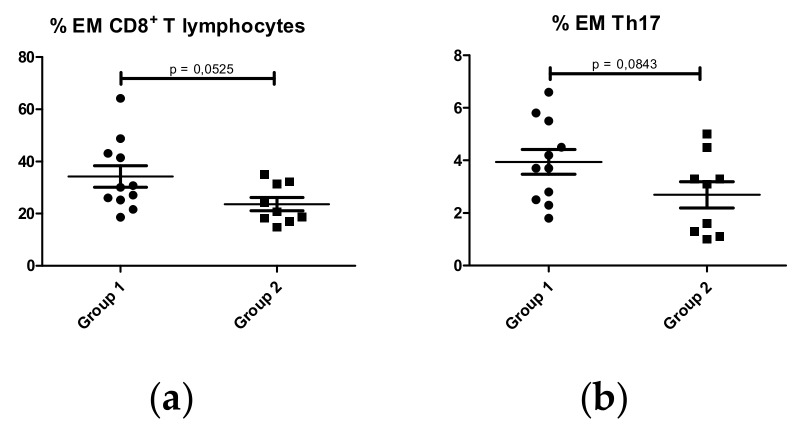

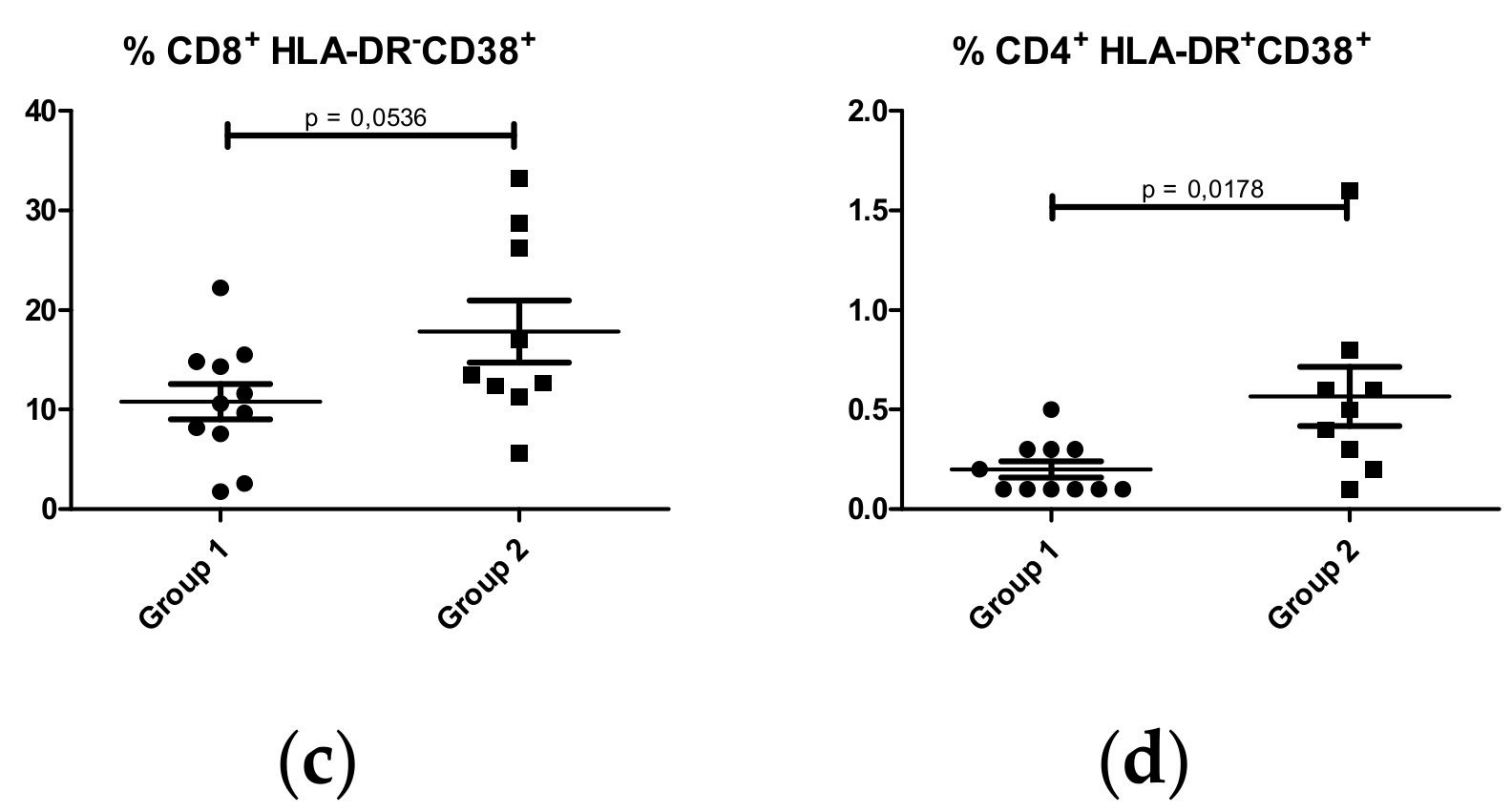
Comparison of percentage of leukocyte subpopulations between the two groups of patients according to CD89^high^ MFI in nonclassical monocytes (CD14^low^CD16^++^): group 1 with CD89^high^ MFI < 4000 (N = 13); group 2 with CD89^high^ MFI > 4000 (N = 9). (**a**) Effector Memory (EM) CD8^+^ T lymphocytes (CD3^+^CD8^+^CCR7^-^CD45RA^-^); (**b**) EM Th17 (CD3^+^CD4^+^CXCR3^-^CCR6^+^CCR7^-^CD45RA^-^); (**c**) activated CD8^+^ T lymphocytes (CD3^+^CD8^+^HLA-DR^-^CD38^+^); (**d**) activated CD4^+^ T lymphocytes (CD3^+^CD4^+^HLA-DR^+^CD38^+^).

Finally, given the observed results of CD89 MFI on nonclassical monocytes, we compared the immunophenotype of leukocyte subpopulations between the two groups of patients. Patients in group 1 had a higher percentage of effector memory CD8^+^ cells (Figure 9a) and a higher percentage of effector memory Th17 lymphocytes (Figure 9b) than patients in group 2, with a lower percentage of activated CD8^+^ and CD4^+^ cells (Figure 9c,d). No significant differences were observed in the other T lymphocyte subpopulations (Figure S4a–k).

No significant differences were observed in B lymphocyte subpopulations, dendritic cell subpopulations and NK lymphocyte subpopulations between the two patient groups (Figure S4l–w).

### 2.3. Serum Levels of Galactose Deficient IgA1

Analysis of serum levels of Gd-IgA1 ELISA in IgAN vs HS showed no significant differences (Patients: 18.44 ng/mL ± 0.1735; HS: 18.12 ng/mL ± 0.1355; p = 0.2133) (Figure S5a)

We also compared Gd-IgA1 levels between the two groups of patients defined on the analysis of CD89 MFI (Group 1 MFI of CD89^high^ on nonclassical monocytes < 4000; Group 2 CD89^high^ MFI on nonclassical monocytes > 4000). Similarly, no significant differences were found between the two groups (18.04 ± 0.232 ng/mL group 1; 18.57 ± 0.272 ng/mL of group 2; p = 0.1672). (Figure S5b).

## 3. Discussion

Although IgAN is the most frequent primary glomerulonephritis and is related to end-stage renal disease in 40% of patients, there are still no specific diagnostic or prognostic biomarkers, which are urgently required.

Until now, no exhaustive analysis of peripheral leukocyte subpopulations has been made in IgAN patients. Our results show that IgAN patients had a different distribution of leukocyte subpopulations compared to HS. Immunophenotyping of leukocyte subpopulations showed that IgAN patients had higher percentages of activated and effector memory T lymphocyte subpopulations, myeloid dendritic cells and CD56^dim^CD16^+^ NK cells than HS. We also found a reduction in CD89 expression on nonclassical monocytes (CD14^low^CD16^++^) in those patients with a more severe renal biopsy and a poor renal function. In line with this, we discerned two groups of IgAN patients in relation to CD89 MFI expression on nonclassical monocytes. Immunophenotype comparison showed that group 1 (lower CD89 expression levels on monocytes) had higher percentages of effector memory CD8^+^ and Th17 CD4^+^ T lymphocytes, and group 2 had higher percentages of activated CD8^+^ and CD4^+^ T lymphocytes. No significant differences were found between the two patient groups or between study subjects and HS with respect to Gd-IgA1 levels.

It is known that monocytes are leukocytes that connect innate immunity with the activation of adaptive immunity. Three subpopulations have been described according to CD14 and CD16 expression, each with different morphological, phenotypical and functional characteristics [14]. Classical monocytes are implicated in acute tissue inflammation and intermediate and nonclassical monocytes in chronic tissue inflammation, favoring angiogenesis and fibrosis [15]. We found that IgAN patients had a trend toward higher percentages of intermediate and nonclassical monocytes, as previously described [16]. These results may correlate with chronic inflammation leading to fibrosis of the glomerular mesangium and end-stage renal disease.

Several studies [17,18] have demonstrated a correlation between Gd-IgA1-sCD89 complex levels and disease progression. The Oxford classification [19] and its posterior validation (Valiga study) [20] include four histological characteristics as independent factors for a worse disease prognosis—mesangial hypercellularity (M1), segmental glomerulosclerosis (S1), endocapillary hypercellularity (E1) and interstitial fibrosis/tubular atrophy (T1). We found a correlation between the lower intensity of CD89 surface expression on nonclassical monocytes with two of the four histological characteristics included in the Oxford classification—S1 and T1, which may reflect increased disease activity, with the deposition of immunocomplexes on the glomerular mesangium, leading to chronic inflammation and tissue fibrosis. Our results suggest that CD89 MFI expression on nonclassical monocytes might be a useful prognostic biomarker to discern IgAN patients with a worse prognosis.

Moreover, effector memory T lymphocyte subpopulations could be prognostic biomarkers combined with CD89 surface expression on nonclassical monocytes, as IgAN patients with a worse prognosis (group 1) had higher percentages of effector memory CD8^+^ and effector memory Th17 CD4^+^ T lymphocytes. These results may suggest the implication of these subpopulations of T lymphocytes in disease activity, probably related to mesangial cell activation (a consequence of Gd-IgA1 and related immune complex deposition), which induces the secretion of IL-6 or TGF-β, two cytokines that induce Th17 polarization.

We observed lower percentages of transitional B lymphocytes and plasmablasts in IgAN patients than in HS, suggesting that most peripheral B lymphocytes are naïve and mature and may also reflect the constant production of antibody-producing plasma cells related to the exhaustion of peripheral blood of its precursors, plasmablasts. To our knowledge, this is the first study to examine the immunological profile of B lymphocyte subpopulations in the peripheral blood of IgAN patients.

Dendritic cells (DC) are a heterogeneous antigen-presenting cell subpopulation which is crucial in initiating and organizing the immune response by inducing the polarization of T lymphocytes. DC subtypes differing in origin, localization and functions have been described. In steady state conditions, DCs are found in secondary lymphoid organs and tissues such as the skin and intestines. Consequently, after an inflammatory stimulus, DCs migrate to the site of inflammation where they are activated and induce T cell polarization according to the inflammatory microenvironment. Our results showed that IgAN patients had higher percentages of myeloid SLAN^-^CD16^+^ DCs than HS, suggesting that these DCs were not inflammatory, as one of the main inflammatory markers described is SLAN [21]. However, IgAN patients with a worse prognosis (group 1) had a trend to higher percentages of myeloid SLAN^+^CD16^+^ DC, suggesting that inflammatory DCs play a role in IgAN pathogenesis, probably contributing to T cell activation and polarization [22], although studies in a higher number of patients are necessary to confirm this.

NK cells are innate immune system leukocytes whose main functions are cell lysis and cytokine production. Two main NK subpopulations have been described according to CD56 and CD16 expression—the CD56^dim^CD16^+^ subpopulation, which represents 90% of NK lymphocytes, and the CD56^bright^CD16^-^ subpopulation [23]. The CD56^dim^CD16^+^ subpopulation has high cytotoxicity compared with the CD56^bright^CD16^-^ subpopulation, which has a higher capacity for cytokine production. Several authors have classified the CD56^bright^CD16^-^ subpopulation as regulatory NK cells [24]. Our results showed that IgAN patients had a higher percentage of the CD56^dim^CD16^+^ subpopulation, suggesting the implication of NK cells in disease pathogenesis through an increase in the inflammatory microenvironment that induces the chemoattraction of more inflammatory cells. Although no differences were observed between IgAN patients, group 1 had a trend toward higher percentages of the CD56^dim^CD16^+^ subpopulation. These results require further investigation.

Recently, Monteiro et al. [25] described the generation of Gd-IgA1 as the first step in the physiopathogenic mechanism of the disease, while in vitro studies by Novak et al. showed that Gd-IgA1 deposition on the glomerular mesangium of the glomerulus alone was not sufficient to induce mesangial cell proliferation [26,27]. Recently, Suzuki et al. [28,29] reported that higher levels of Gd-IgA1 and of related immune complexes were associated with disease activity and allowed the differentiation of IgAN from other renal diseases. In line with these authors, we found a trend toward higher Gd-IgA1 levels in IgAN patients compared with HS. However, higher Gd-IgA1 concentrations have also been found in healthy relatives of patients compared to HS, suggesting the test by itself has low specificity. Therefore, it has been proposed to combine the determination of Gd-IgA1 levels with other biomarkers [29].

In conclusion, although our results should be confirmed in a larger and independent cohort of patients, they support the hypothesis that the reduction in CD89 expression on monocytes, together with higher serum levels of Gd-IgA1 and higher percentages of inflammatory leukocyte subpopulations, increases diagnostic and prognostic sensitivity of IgAN.

## 4. Materials and Methods

### 4.1. Patients

A total of 22 biopsy-proven IgAN patients and 12 healthy subjects (HS) were included. All subjects gave their informed consent for inclusion before they participated in the study. The study was conducted in accordance with the Declaration of Helsinki, and the protocol was approved on the 7th of March of 2017 by the Ethics Committee of Germans Trias i Pujol Hospital (Project Identification code: PI-17-013). The main demographic and clinical features of patients are summarized in Table 2. Renal function was defined by estimated glomerular filtration rate (eGFR) (mL/min/1.73m^2^), serum creatinine levels (mg/dL), proteinuria (proteinuria/creatinine ratio) and hematuria (defined as ≥ 3 red blood cells per high power cortical field). The Oxford classification was used for the histological classification of the renal biopsy. No patients had received corticosteroids or immunosuppressive agents at study entry.

### 4.2. Immunological Characterization and Flow Cytometric Analysis

Peripheral blood samples were collected in ethylene diamine tetra acetic acid (EDTA). Samples of whole blood were processed between 2-4 h after extraction. Samples were incubated with monoclonal antibodies for 20 min at room temperature and in darkness. After erythrocyte lysis, samples were washed and acquired on a LSRFortessa flow cytometer (BD Biosciences, San José, CA, USA). The analysis strategy selected the subpopulation using SSC/CD45 staining, what allowed for the exclusion of debris. For each panel we used a stopping gate for the reference population, excluding debris—(1) B-cell panel, minimum 20.000 events of CD19^+^ cells; (2) T cell panel, minimum 50.000 events of CD3^+^ cells; (3) Treg panel, minimum 20.000 events of CD4^+^ cells; (4) dendritic cell and NK cell panel, minimum 100.000 events of PBMCs; 5) monocytes panel, minimum 20.000 events of CD14^+^ cells. Data analysis was performed by FACSDiva software (BD Biosciences).

Leukocyte subpopulations were defined using the following monoclonal antibodies per panel—(i) T cell panel: CD3-V450, CD4-PercP-Cy5.5, CD45RA PE-Cy7, CCR7 PE, CD38 APC, CD8 APC-H7, HLA-DR V500 (BD Bioscience), CD45 AF700 (Biolegend, San Diego, CA, USA; (ii) Treg panel: CD4 PerCP-Cy5.5, CD25 PE, CCR4 PE-Cy7, CD127 AF647, CD45RO APC-H7, CD3 V450, HLA-DR V500 (BD Bioscience) and CD45 AF700 (Biolegend; (iii) B cell panel: CD24 FITC, CD19 PerCP-Cy5.5, CD38 APC, CD20 APC-H7, CD3 V500 (BD Bioscience), IgD PE-Cy7, CD27 BV421 and CD45 AF700 (Biolegend); (iv) dendritic cell and NK cell panel: CD3+CD19 APC-H7, CD56 PE, CD16 APC, CD14 V450, CD123 PerCP-Cy5.5, CD11c PE-Cy7, HLA-DR V500 (BD Biosciemce) and Slan FITC (Milteny Biotec, Bergisch Gladbach, Germany); (ivi) monocytes panel: CD15-APC (BD-Biosciences), CD14-APCH7, CD45-AF700, CD16-V450 and CD89-PE. Leukocyte subpopulations (T cell panel, B cell panel, Treg panel and dendritic cell and NK cell panel) were analyzed using standardized European protocol ENTIRE HIP-C [30,31], which analyses markers of activation and maturation of all leukocyte subpopulations. The gating strategy to analyse these leukocyte subpopulations was performed as previously described in our group [32]. In relation to the monocytes panel, we designed a flow cytometry panel to measure CD89 expression on classical monocytes (CD14^+^CD16^-^), intermediate monocytes (CD14^+^CD16^+^) and nonclassical monocytes (CD14^low^CD16^+^). The gating strategy of this panel is represented in Figure S6.

### 4.3. Serum Levels of Galactose Deficient IgA1

Serum levels of galactose-deficient IgA1 were measured by ELISA (Galactose-deficient IgA1 Assay kit-IBL, Immuno-Biological Laboratories Co. Japan) following the manufacturer’s instructions and using a monoclonal antibody specific for galactose-deficient IgA1 by Suzuki et al [33].

### 4.4. Statistical Analysis

Continuous data were expressed as mean ± standard deviation (SD) and statistical significance for intergroup differences was assessed by Student’s t-test. CD89 expression was stratified according to the best threshold chosen using the Youden index. The Receiver Operating Characteristic (ROC) curve was used to divide IgAN patients and the best cut-off in terms of sensitivity and specificity was identified. The results are shown as the area under the curve (AUC) and the corresponding confidence intervals. The statistical analysis was made using Graphpad prism 5.0. A two-tailed p-value of <0.05 was considered statistically significant.

## 5. Conclusions

The results of this preliminary study show the participation of the innate and adaptive immune systems in the pathogenesis of IgAN patients. Constant cooperation between them is essential for the correct activity of the immune system. We found differences in CD89 surface intensity on nonclassical monocytes and in the percentage of leukocyte subpopulations between the two groups of IgAN patients. Taken together, the combination of these two parameters, once validated in a large cohort of patients, might be useful in clinical practice as a predictor of the disease prognosis.

## Figures and Tables

**Table 1:** Renal biopsy characteristics of the two group of patients according to CD89^high^ MFI on nonclassical monocytes (CD14^low^CD16^++^).

	Group 1 (N = 13) CD89^high^ MFI < 4000	Group 2 (N = 9) CD89^high^ MFI > 4000	P Value
Oxford			
M1	13 (100%)	9 (100%)	-
S1	10 (76.92%)	2 (22.22%)	0.0274
E1	2 (15.38%)	2 (22.22%)	1.000
T1-2	7 (53.84%)	1 (11.11%)	0.0669

Data are expressed as percentages. M1, mesangial hypercellularity presence; S1, segmental glomerulosclerosis; E1, endocapillary hypercellularity presence; T1-2, interstitial fibrosis/tubular atrophy (IFTA).

**Table 2:** Demographic, clinical, and biochemical characteristics of IgAN patients.

	IgAN Patients (N = 22)	Normality Range
Gender (Male), n (%)	14 (63.63%)	-
Age, years	50.63	-
IgA, mg/dL	368.55 ± 228.39	70–400
C3, mg/dL	116.41 ± 9.89	90-180
C4, mg/dL	27.78 ± 1.41	10–40
eGFR, mL/min/1,73m^2^	53 ± 26.16	>90
Serum creatinine, mg/dL	1.76 ± 3.03	0.72–1.18
Proteinuria, mg/g creatinine)	1212.14 ± 1811.89	0–200
Hematuria	7 ± 15.90	<3
Oxford classification		
M1	22 (100%)	-
S1	12 (54.54%)	-
E1	4 (18.18%)	-
T1-2	7 (31.82%)	-

Data are expressed as mean ± DS. eGFR, estimated glomerular filtration rate; M1, mesangial hypercellularity; S1, segmental glomerular sclerosis; E1, endocapillary hypercellularity; T1-2, interstitial fibrosis/tubular atrophy (IFTA); C3 and C4, soluble complement fragment.

## Supplementary Materials

Supplementary materials can be found at https://www.mdpi.com/1422-0067/21/4/1389/s1.
