# Supplementary material for: Immunological Pattern in IgA Nephropathy
Source: Int J Mol Sci. 2020 Feb 18;21(4):1389. doi: 10.3390/ijms21041389 (PMC7073027; doi:10.3390/ijms21041389)

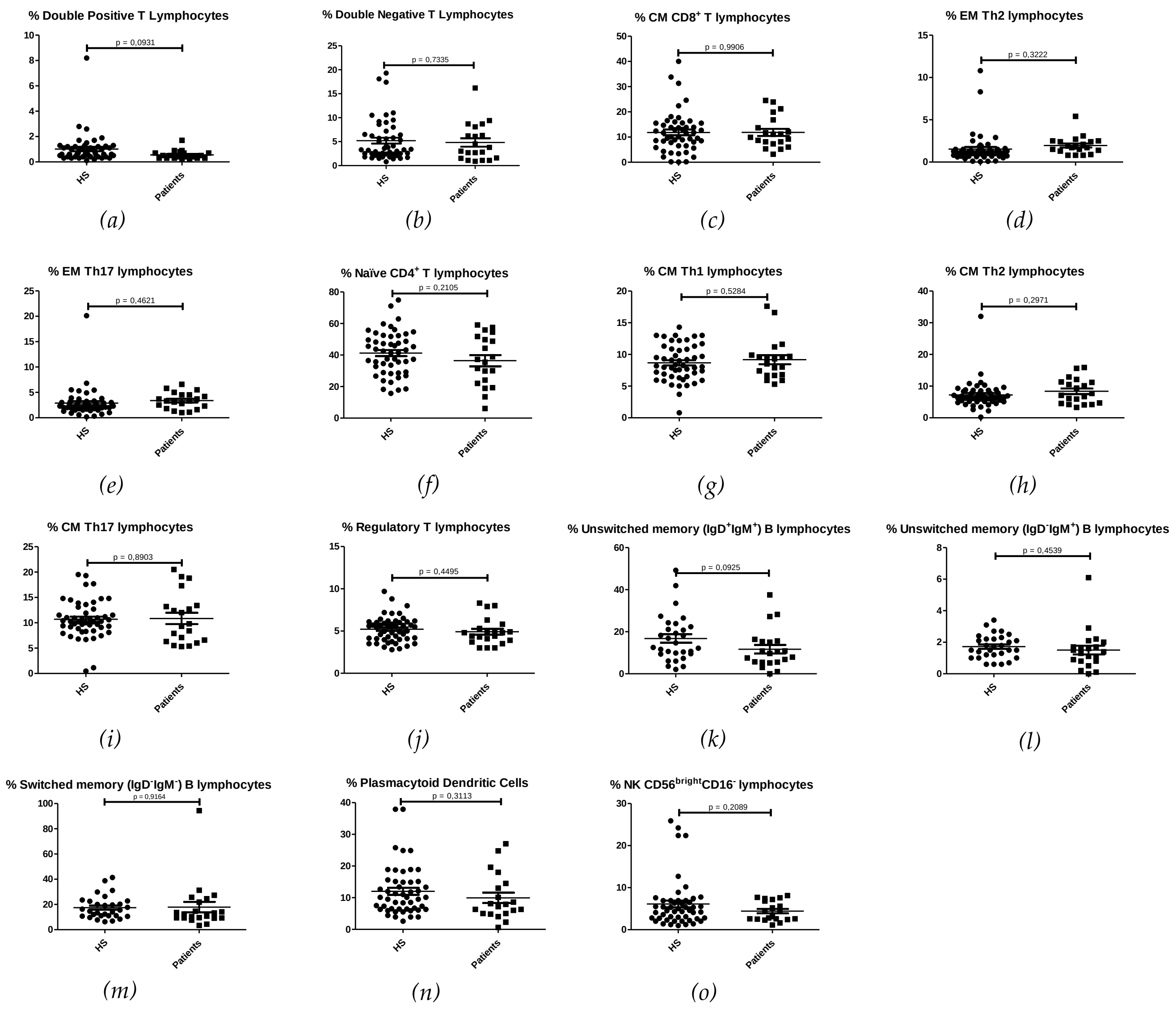

Figure S1: Comparison of percentages of leukocyte subpopulations in peripheral blood between IgAN patients (N=22) and HS (N=50). **(a)** Double positive ($CD4^+CD8^+$) T lymphocytes; **(b)** Double negative ($CD4^-CD8^-$) T lymphocytes; **(c)** CM $CD8^+$ T lymphocytes ($CCR7^+CD45RA^-$); **(d)** EM Th2 lymphocytes ($CD3^+CD4^+CXCR3^-CCR6^-CCR7^-CD45RA^-$); **(e)** EM Th17 lymphocytes ($CD3^+CD4^+CXCR3^-CCR6^+CCR7^-CD45RA^-$); **(f)** Naïve $CD4^+$ T lymphocytes ($CD3^+CD4^+CCR7^+CD45RA^+$) **(g)** CM Th1 lymphocytes ($CD3^+CD4^+CXCR3^+CCR6^-CCR7^+CD45RA^-$); **(h)** CM Th2 lymphocytes ($CD3^+CD4^+CXCR3^-CCR6^-CCR7^+CD45RA^-$); **(i)** CM Th17 lymphocytes ($CD3^+CD4^+CXCR3^-CCR6^+CCR7^+CD45RA^-$); **(j)** Regulatory T lymphocytes; **(k)** unswitched memory B lymphocytes ($CD19^+CD27^+IgD^+IgM^+$); **(l)** unswitched memory B lymphocytes ($CD19^+CD27^+IgD^-IgM^+$); **(m)** switched memory B lymphocytes ($CD19^+CD27^+IgD^-IgM^-$); **(n)** plasmacytoid dendritic cells ($HLA\text{-}DR^+CD11c^-CD123^+$); **(o)** NK $CD56^{bright}CD16^-$ lymphocytes

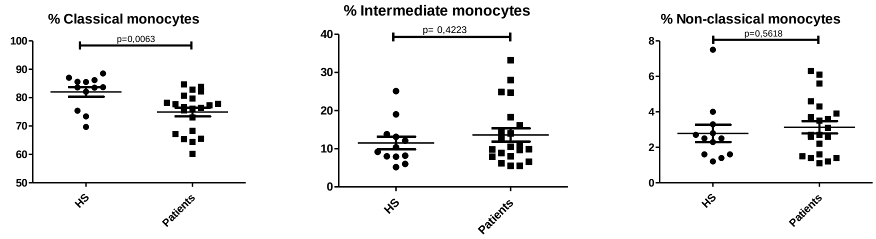

(a) *(b)* *(c)*

Figure S2: Comparison of monocyte subsets distribution between IgAN patients (N=22) and HS (N=12). (a) Percentages of classical monocytes ($CD14^{+}CD16^{-}$); **(b)** percentages of intermediate monocytes ($CD14^{+}CD16^{+}$); **(c)** percentages of non-classical monocytes ($CD14^{low}CD16^{++}$)

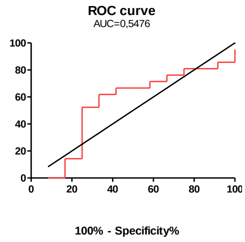

Figure S3: Discriminatory capacity of CD89 MFI on non-classical monocytes ($CD14^{low}CD16^{++}$). The optimal cut-off was 4000; AUC: Area Under the Curve

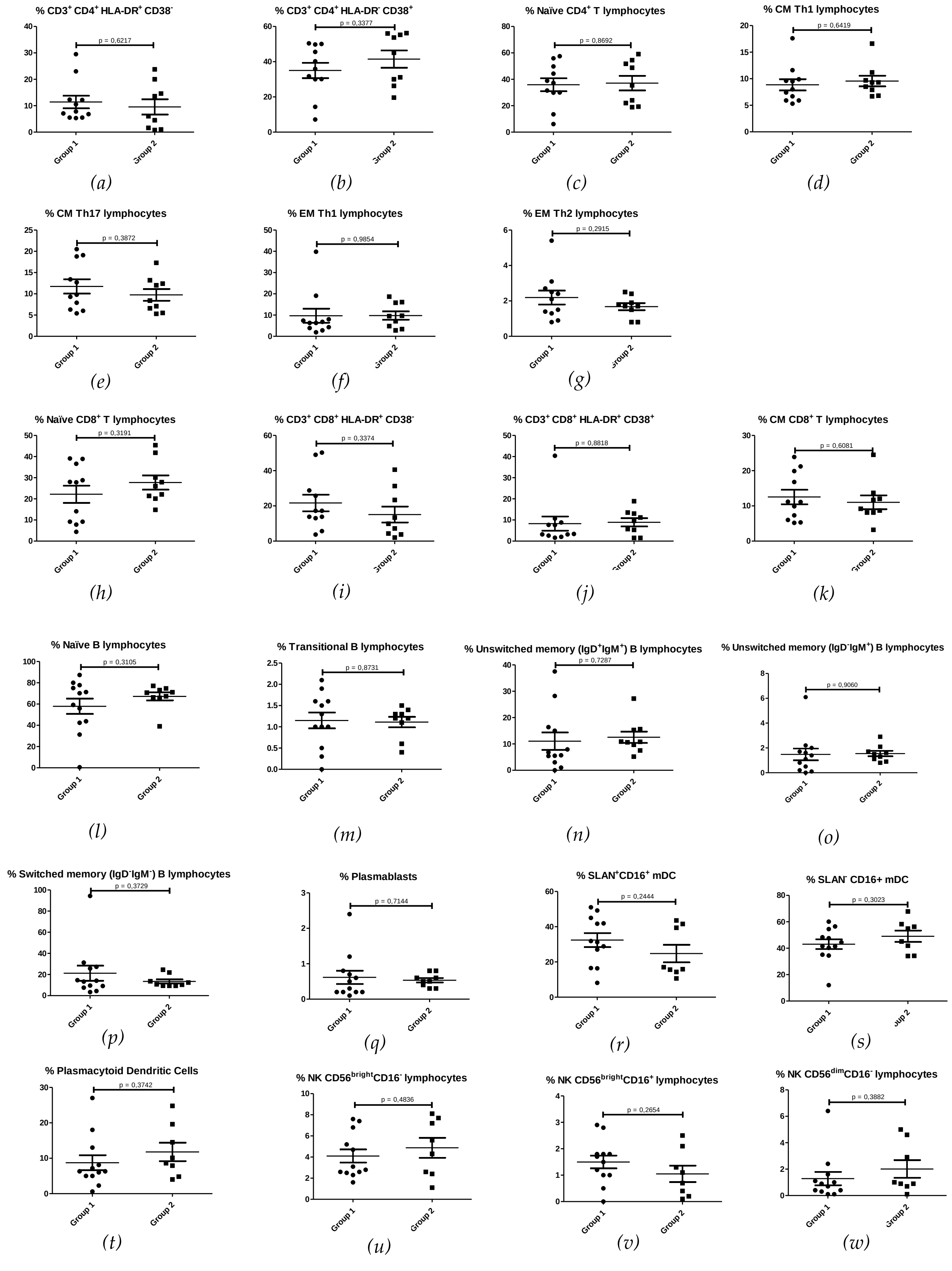

% CD3+ CD4+ HLA-DR+ CD38-
p = 0,6217
(a)
% CD3+ CD4+ HLA-DR- CD38+
p = 0,3377
(b)
% Naïve CD4+ T lymphocytes
p = 0,8692
(c)
% CM Th1 lymphocytes
p = 0,6419
(d)
% CM Th17 lymphocytes
p = 0,3872
(e)
% EM Th1 lymphocytes
p = 0,9854
(f)
% EM Th2 lymphocytes
p = 0,2915
(g)
% Naïve CD8+ T lymphocytes
p = 0,3191
(h)
% CD3+ CD8+ HLA-DR+ CD38-
p = 0,3374
(i)
% CD3+ CD8+ HLA-DR+ CD38+
p = 0,8818
(j)
% CM CD8+ T lymphocytes
p = 0,6081
(k)
% Naïve B lymphocytes
p = 0,3105
(l)
% Transitional B lymphocytes
p = 0,8731
(m)
% Unswitched memory (IgD+IgM+) B lymphocytes
p = 0,7287
(n)
% Unswitched memory (IgD-IgM+) B lymphocytes
p = 0,9060
(o)
% Switched memory (IgD-IgM-) B lymphocytes
p = 0,3729
(p)
% Plasmablasts
p = 0,7144
(q)
% SLAN+CD16+ mDC
p = 0,2444
(r)
% SLAN- CD16+ mDC
p = 0,3023
(s)
% Plasmacytoid Dendritic Cells
p = 0,3742
(t)
% NK CD56brightCD16- lymphocytes
p = 0,4836
(u)
% NK CD56brightCD16+ lymphocytes
p = 0,2654
(v)
% NK CD56dimCD16- lymphocytes
p = 0,3882
(w)
Group 1
Group 2

Figure S4: Comparison of percentage of leukocyte subpopulations between the two groups of patients according to CD89^{high} MFI in non-classical monocytes (CD14^{low}CD16^{++}): group 1 with CD89^{high} MFI < 4000 (N=13); group 2 with CD89^{high} MFI > 4000 (N=9). **(a)** Activated CD4^{+} T lymphocytes (CD3^{+}CD4^{+}HLA-DR^{+}CD38^{-}); **(b)** Activated CD4^{+} T lymphocytes (CD3^{+}CD4^{+}HLA-DR^{-}CD38^{+}); **(c)** Naïve CD4^{+} T lymphocytes (CD3^{+}CD4^{+}CCR7^{+}CD45RA^{+}); **(d)** Central Memory (CM) Th1 lymphocytes (CD3^{+}CD4^{+}CXCR3^{+}CCR6^{-}CCR7^{+}CD45RA^{-}); **(e)** Central Memory (CM) Th17 lymphocytes (CD3^{+}CD4^{+}CXCR3^{-}CCR6^{+}CCR7^{+}CD45RA^{-}); **(f)** Effector Memory (EM) Th1 lymphocytes (CD3^{+}CD4^{+}CXCR3^{+}CCR6^{-}CCR7^{-}CD45RA^{-}); **(g)** Effector Memory (EM) Th2 lymphocytes (CD3^{+}CD4^{+}CXCR3^{-}CCR6^{-}CCR7^{-}CD45RA^{-}); **(h)** Naïve CD8^{+} T lymphocytes (CCR7^{+}CD45RA^{+}); **(i)** Activated CD8^{+} T lymphocytes (CD3^{+}CD8^{+}HLA-DR^{+}CD38^{-}); **(j)** Activated CD8^{+} T lymphocytes (CD3^{+}CD8^{+}HLA-DR^{+}CD38^{+}); **(k)** Central Memory (CM) CD8^{+} T lymphocytes (CCR7^{+}CD45RA^{-}); **(l)** Naïve B lymphocytes (CD19^{+}CD27^{-}IgD^{+}IgM^{+}); **(m)** Transitional B lymphocytes (CD19^{+}CD27^{-}CD24^{high}CD38^{high}); **(n)** Unswitched memory B lymphocytes (CD19^{+}CD27^{+}IgD^{+}IgM^{+}); **(o)** Unswitched memory B lymphocytes (CD19^{+}CD27^{+}IgD^{-}IgM^{+}); **(p)** Switched memory B lymphocytes (CD19^{+}CD27^{+}IgD^{-}IgM^{-}); **(q)** Plasmablasts (CD19^{+}CD27^{+}CD20^{-}CD38^{high}); **(r)** SLAN^{+}CD16^{+} myeloid Dendritic Cells (mDC); **(s)** SLAN^{-}CD16^{+} myeloid Dendritic Cells (mDC); **(t)** Plasmacytoid Dendritic Cells (HLA-DR^{+}CD11c^{-}CD123^{+}); **(u)** NK CD56^{bright}CD16^{-} Lymphocytes; **(v)** NK CD56^{bright}CD16^{+} lymphocytes; **(w)** NK CD56^{dim}CD16^{-} lymphocytes

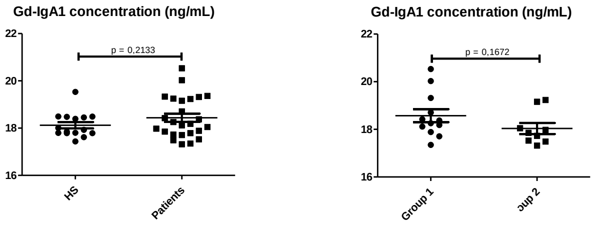

(a) *(b)*

Figure S5: Comparison of serum Gd-IgA1 concentration (ng/mL). (a) Between HS (N=12) and patients (N=22); **(b)** Between the two groups of IgAN patients according to $CD89^{high}$ MFI on non-classical monocytes ($CD14^{low}CD16^{++}$), group 1 MFI of $CD89^{high}$ on non-classical monocytes<4000; Group 2 $CD89^{high}$ MFI on non-classical monocytes>4000

Figure S6: Gating strategy for monocyte subpopulations analysis and CD89 expression.

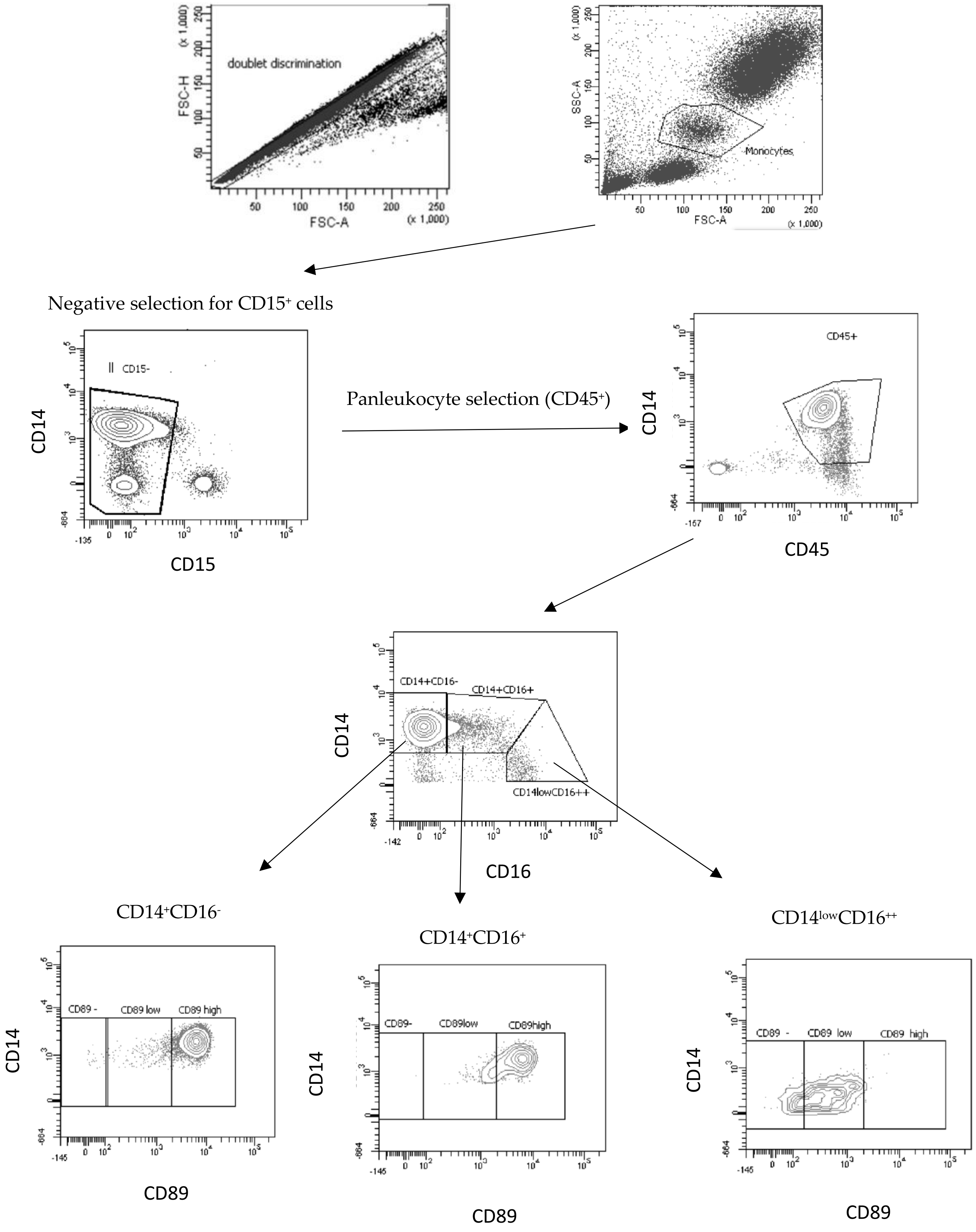

Supplement: Supplementary file 1 [file ijms-21-01389-s001.pdf]
